# Evaluation of efficacy and safety of endovascular coiling for patients with aneurysmal subarachnoid hemorrhage: A protocol for systematic review and meta-analysis

**DOI:** 10.1097/MD.0000000000025728

**Published:** 2021-05-21

**Authors:** Qiu-Rong Fu, Yan Wang, Shi-Bu Lin, Yan Yang

**Affiliations:** Department of Neurosurgery, The First Affiliated Hospital of Hainan Medical University, Haikou, Hainan, PR China.

**Keywords:** aneurysmal subarachnoid hemorrhage, efficacy, endovascular coiling, safety, systematic review

## Abstract

**Background:**

There is an elevated risk of rebleeding when the aneurysm is left untreated in patients diagnosed with aneurysmal subarachnoid hemorrhage (SAH). Occlusion of the lumen of the aneurysm using endovascular coiling is a common method to prevent rebleeding by occluding the aneurysm. This study aims to evaluate the efficacy and safety of endovascular coiling in patients with aneurysmal SAH.

**Methods:**

A systematic search for relevant articles will be performed in 4 English electronic databases, including MEDLINE (from 1966 to October 2020), EMBASE (from 1980 to October 2020), the Cochrane Library (from 2020, issue 10), Scopus (from 1823 to October 2020), and 3 Chinese electronic databases, including Chinese Biomedical Literature Database (from 1995 to October 2020), WanFang (last searched October 2020), and China National Knowledge Infrastructure (last searched October 2020). This study will comprise randomized controlled trials (RCTs) that evaluate the effectiveness and safety of using endovascular coiling in the treatment of aneurysmal SAH. The articles in the databases will be independently screened by 2 authors to select potential studies, extract data, and evaluate the bias risk in the selected studies. This study will use suitable statistical methods to merge result data.

**Results:**

The results of this study will be useful in determining the efficacy and safety of endovascular coiling for treating patients with aneurysmal SAH.

**Conclusion:**

The findings of this study will summarize the most recent evidence on the effectiveness and safety of using endovascular coiling to treat aneurysmal SAH.

**Ethics and dissemination:**

The present work does not involve any humans or animals; therefore, ethical approval is not needed.

**Systematic review registration:**

December 2, 2020.osf.io/yj4gq (https://osf.io/yj4gq/)

## 1 Introduction

Subarachnoid hemorrhage (SAH) is categorized under strokes. According to population-based studies, it is prevalent in approximately 9 out of 100,000 people each year, and this includes fatalities outside hospitals. However, there are some regional variations across the world.^[1,2]^ SAH is mostly prevalent in Finland and Japan, the former reports 19.7 per 100,000 people, and the latter reports 22.7 cases per 100,000 citizens, respectively.^[2]^ Aneurysmal SAH can cause several severe conditions, and patients could undergo cognitive decline, cerebral vasospasm, and delayed cerebral ischemia.^[3,4]^ Generally, a headache is the most commonly encountered symptom, and patients describe this headache as the worst they have ever experienced, it is abrupt and peaks in intensity in a maximum of 1 hour.^[5]^ One third of all people become a fatality within 3 months of the hemorrhage, meanwhile, 1 in 5 individuals are indisposed and depends on others to care for them and help them with their daily activities.^[6]^ Due to the poor clinical outcomes following a hemorrhage and considering the young age at which it occurs, the loss of productive human life from aneurysmal SAH is identical to the loss caused by ischemic strokes, which is a highly prevalent type of stroke.^[7–9]^ Considering the distinct anatomical characteristics and the relative infrequency of aneurysmal SAH, an optimal management strategy is yet to be found. In the past, neurosurgical clipping of the aneurysm has been the standardized method to avert recurrent hemorrhage. However, the advent of detachable coils, endovascular coiling has become more common for treating the aneurysm. Clinical practitioners have replaced neurosurgical clipping with endovascular coiling as their preferred method of treatment, especially if coiling is technically feasible. In light of this, the present study is performed to evaluate the efficacy and safety of endovascular coiling as a therapeutic method for patients with aneurysmal SAH.

## 2 Methods

### 2.1 Registration

The present systematic review protocol has been registered on the Open Science Framework (OSF, http://osf.io/). The registration DOI number is 10.17605/OSF.IO/YJ4GQ. The content of this protocol is in accordance with the Preferred Reporting Items for Systematic Review and Meta-Analyses Protocols (PRISMA-P) statement guidelines.

### 2.2 Eligibility criteria for included studies

#### 2.2.1 Types of studies

This study comprises randomized controlled trials (RCTs) that evaluate the efficacy and safety of endovascular coiling in patients with aneurysmal SAH. Case reports, reviews, conferences, non-RCTs, and studies involving animals are excluded.

#### 2.2.2 Types of participants

Regardless of age and gender, this study will involve patients who exhibited symptoms of aneurysmal SAH with aneurysmal pattern or computerized tomography scan, or an aneurysm indicated by angiography.

#### 2.2.3 Types of interventions

Each participant in the experimental group must be administered endovascular coiling treatment as the experimental intervention. Meanwhile, each participant in the control group can either receive neurosurgical clipping, no intervention, placebo, or an alternative intervention method as the control intervention.

#### 2.2.4 Types of outcome measures

##### 2.2.4.1 Major outcomes

The major outcomes include death from any cause, or dependence in daily activities (defined as Glasgow Outcome Scale, and poor clinical outcome was defined as Glasgow Outcome Scale one-three).^[10]^

##### 2.2.4.2 Minor outcomes

The minor outcomes include:

1. delayed cerebral ischemia;
2. recurrent hemorrhage;
3. complications from the intervention.

### 2.3 Search methods for identification of studies

#### 2.3.1 Electronic searches

We will perform a systematic search of related articles in electronic databases, including MEDLINE (from 1966 to October 2020), EMBASE (from 1980 to October 2020), the Cochrane Library (from 2020, issue 10), Scopus (from 1823 to October 2020), Chinese Biomedical Literature Database (from 1995 to October 2020), WanFang (last searched October 2020), and China National Knowledge Infrastructure (last searched October 2020). This study includes RCTs that evaluate the effectiveness and safety of endovascular coiling as a therapeutic strategy for aneurysmal SAH. Languages were restricted to English and Chinese. The following MeSH terms, free text, related synonym, and their combinations to search databases are used: “subarachnoid hemorrhage,” coiling∗, aneurysm∗, intracranial∗, “randomized controlled trial,” “randomized controlled trial,” randomly∗, and RCT∗.

#### 2.3.2 Searching other resources

This study will also search for extra relevant published, unpublished, or ongoing trials. The trialists will be contacted. Besides, the reference lists of all relevant publications are included in the search.

### 2.4 Search methods for identification of studies

#### 2.4.1 Selection of studies

**Figure 1:**
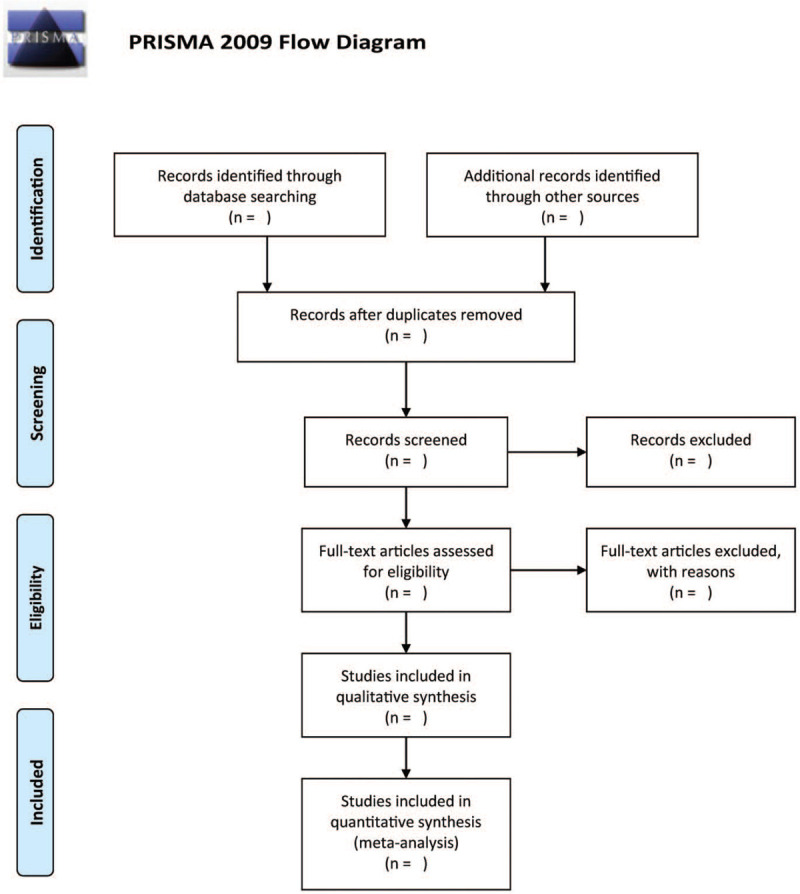
The research flowchart.

The potential studies from the search will be reviewed by 2 authors independently to assess their relevance according to the criteria to select a study. Any form of disagreement will be resolved through discussion. Figure 1 illustrates the detailed process of selecting studies.

#### 2.4.2 Data extraction and management

Relevant trial data will be extracted by 2 authors independently using a prepiloted form to extract data. The RevMan 5.3 software is used to store and manage the extracted data. All disagreements will be resolved through discussion.

#### 2.4.3 Assessment of risk of bias in included studies

Based on the Cochrane Collaboration “Risk of bias” tool, 2 authors will independently evaluate the risk of bias in the included studies.^[11]^

#### 2.4.4 Measures of treatment effect

The risk ratio with 95% confidence interval will be used to estimate the main outcome measures.

#### 2.4.5 Assessment of heterogeneity

This study will use Cochrane Q test and *I*^*2*^ to assess the heterogeneity of the outcomes. Significant heterogeneity is implied if the *P* value exceeds .1 or if *I*^*2*^ is less than 50%, in which case, a fixed-effects model is used to merge the results; else, the random-effects model is used to merge the results.

#### 2.4.6 Assessment of publication bias

If the number of included studies exceed 10, funnel plots will be used to assess the risk of publication bias.

#### 2.4.7 Sensitivity analysis

A sensitivity analysis will be utilized to assess the stability of our findings. It will exclude studies with high bias risk or unclear methodological data.

## 3 Discussion

Aneurysmal SAH is a fatal condition. In addition to affecting the brain, it also affects many other vital organ systems.^[12]^ Admittedly, over the recent years, there has been a stable reduction of mortality related to aneurysmal SAH, and the number has decreased from over 50% to about 35%; however, it is still related to considerable morbidity and mortality. Occlusion of the lumen of the aneurysm using endovascular coiling has been commonly used to prevent rebleeding by occluding the aneurysm. More recently, an increasing number of researchers have explored the effects of endovascular coiling as a therapeutic strategy for aneurysmal SAH. However, the results of these studies are not conclusive. Therefore, the present study is performed to evaluate the efficacy and safety of endovascular coiling in patients with aneurysmal SAH. This study provides updated evidence of endovascular coiling for treating patients with aneurysmal SAH.

## Author contributions

**Conceptualization:** Qiu-Rong Fu.

**Data curation:** Qiu-Rong Fu, shibu lin.

**Formal analysis:** Qiu-Rong Fu, Yan Wang.

**Funding acquisition:** Yan Wang, Yan Yang.

**Investigation:** Qiu-Rong Fu.

**Methodology:** Qiu-Rong Fu.

**Project administration:** Yan Wang.

**Resources:** shibu lin.

**Software:** Qiu-Rong Fu, shibu lin.

**Supervision:** Yan Wang.

**Validation:** shibu lin, Yan Yang.

**Visualization:** Qiu-Rong Fu, Yan Wang.

**Writing – original draft:** Qiu-Rong Fu.

**Writing – review & editing:** shibu lin, Yan Yang.
